# Extracellular and Intracellular Bacterial Compounds in the Synthesis of Inorganic Particles: Silver Nanoparticles

**DOI:** 10.3390/ijms27167150

**Published:** 2026-08-10

**Authors:** Svetlana Konnova, Svetlana Batasheva, Ilnur Ishmukhametov, Anna Stavitskaya, Kirill Cherednichenko, Elvira Rozhina

**Affiliations:** 1 Institute of Fundamental Medicine and Biology, Kazan Federal University, 420008 Kazan, Republic of Tatarstan, Russia; svbatasheva@gmail.com (S.B.); irishmukhametov@gmail.com (I.I.); 2 Department of Physical and Colloid Chemistry, Gubkin Russian State University of Oil and Gas, 119991 Moscow, Russia; stavitsko@mail.ru (A.S.); cherednichenko.k@gubkin.ru (K.C.)

**Keywords:** bacterial filtrate, cell-free filtrate, *Serratia marcescens*, *Escherichia coli*, biosynthesis, silver nanoparticles, antimicrobial activity

## Abstract

The primary objective of this study was to compare the contribution of cellular and cell-free filtrates of *Serratia marcescens* ATCC 9986 to the synthesis of silver nanoparticles, followed by a determination of the inhibitory activity on growth of *S. marcescens* and *E. coli* of the obtained biogenic silver nanoparticles. The synthesized silver nanoparticles exhibited maximum absorption at 257 nm and 262 nm when analyzed by ultraviolet–visible spectroscopy (UV–Vis). Energy-dispersive X-ray spectroscopy (EDX) analysis revealed a peak at 3 keV, corresponding to silver nanocrystals. Characterization by TEM, EDX, and IR spectroscopy demonstrated the presence of organic compounds in the nanoparticles. IR analysis revealed the presence of proteins, carbohydrates, carboxylic acids, and other compounds with saturated and unsaturated C-C bonds in the nanoparticles. Silver nanoparticles synthesized using cell-free and cellular filtrates inhibited growth of antibiotic-resistant bacteria *E. coli*. Moreover, cell-free filtrate of *S. marcescens* exerted inhibitory activity against *E. coli*, but not against its source, *S. marcescens* bacteria.

## 1. Introduction

The increasing distribution of multidrug-resistant (MDR) pathogens necessitates the search for new effective antimicrobial agents. Nanotechnology methods make it possible to create new antimicrobial materials against antibiotic-resistant pathogens. Among such materials, silver nanoparticles are highly praised for their large surface area, antibacterial and optical properties [1]. Numerous studies have shown that silver has antimicrobial [2], antiviral [3,4], anti-inflammatory [5], antiangiogenic [6], and antitumor [7] activities. The antiplasmodial [8], larvicidal [9], leishmanicidal [10], and scolicidal [11] effects of silver are also well known. Silver nanoparticles are capable of continuously releasing silver ions, which is considered one of the mechanisms of their antimicrobial action. Silver ions can be adsorbed on the bacterial cell surface due to electrostatic attraction and affinity for sulfur-containing proteins. Adsorbed ions can increase the permeability of the cell wall and cytoplasmic membrane and lead to the destruction of the bacterial membrane. The penetration of ions into the cell can lead to the deactivation of respiratory enzymes in the electron transport chain through the formation of reactive oxygen species and subsequently to the inhibition of adenosine triphosphate synthesis. Also, reactive oxygen species can be a major factor causing damage to the cell membrane and changes in deoxyribonucleic acid (DNA). The interaction of silver ions with sulfur and phosphorus in DNA can disrupt DNA replication and cell division, ultimately leading to the death of microorganisms. Additionally, silver ions can inhibit protein synthesis by destroying ribosomes in the cytoplasm. In addition to their ability to release ions, silver nanoparticles can also directly kill bacteria. Upon contact with the cell surface, silver nanoparticles can concentrate in its recesses and can subsequently lead to its destruction. Silver nanoparticles have the ability to penetrate bacterial cell walls and subsequently alter the structure of the cell membrane due to their size. Disruption of the integrity of the cytoplasmic membrane can lead to the destruction of organelles and cell lysis. Furthermore, silver nanoparticles can affect signaling in bacteria by dephosphorylating tyrosine residues on peptide substrates. Disruption of signaling can lead to cell apoptosis and the cessation of cell proliferation [12,13,14,15,16].

Silver nanoparticles are successfully synthesized by biological methods (green synthesis) using plants, fungi, and bacteria [17,18,19]. In the biological synthesis, organic compounds formed by living objects act as reducing and stabilizing agents. This approach minimizes the use of surfactants, synthetic polymers, solvents, and toxic reducing agents [16,20,21,22]. Green nanoparticle synthesis methods are considered environmentally friendly.

Among biological synthesis methods, bacterial nanoparticle synthesis is the most effective, as bacteria are abundant, have a high growth rate, and quickly adapt to extreme environmental conditions. Some bacteria have been shown to grow successfully in the presence of high concentrations of metal ions [23]. Bacteria are easily cultivated, and their cultivation is economically viable. Furthermore, some enzymatic systems of bacteria promote reduction of metal ions [24].

Substances produced by bacterial cells are often applied for the biosynthesis of silver nanoparticles [25,26,27]. Bacterial lysates are rich in molecules with various biological activities (antibiotics, enzymes), which can serve as both reducing and oxidizing agents in the formation of metal nanoparticles [28]. Several studies have shown that bacteria can reduce metal ions to metal nanoparticles both intracellularly and extracellularly. The mechanisms of these synthetic reactions vary. Extracellular synthesis of metal nanoparticles is usually accomplished through ion reduction by compounds secreted by cells into the environment [26,29,30]. Proteins and compounds with aldehyde and keto groups present in cell-free culture fluid are involved in the reduction of metal ions [24,28,31]. Cell-secreted enzymes such as NADH-dependent reductase, nitrate reductase, and sulfur-containing proteins can reduce Ag^+^ ions to Ag^0^, leading to the formation of silver nanoparticles [32,33]. In the intracellular synthesis, silver ions penetrate into the microorganisms and bind either to reductase enzyme or to similar proteins, which leads to the reduction of Ag^+^ to Ag^0^, followed by the formation of nanoparticles in the cytoplasm [32,34].

There are studies on the production of silver nanoparticles using bacterial compounds [30,35,36]. However, most studies examined the involvement of either bacterial supernatant or cell lysate in nanoparticle synthesis. In all of these studies, the nanoparticles produced were not tested against their source organism. Thus, the question of whether nanoparticles produced using bacterial extracts or supernatants can be used to combat the source bacterium has remained unanswered until now. Moreover, the field of green nanoparticle synthesis is rapidly growing because researchers are seeking the simplest and therefore the least resource-intensive methods for producing silver nanoparticles. Extracellular nanoparticle synthesis is a more cost-effective technique than synthesis using cellular extracts because the latter also requires a cell lysis step. Therefore, it is important to test whether similar or more active nanoparticles against the target bacteria can be produced using the more efficient extracellular synthesis approach.

In this work, the biosynthesis of silver nanoparticles using *Serratia marcescens* bacteria has been studied. The genus Serratia is widely used in the pharmaceutical industry, microbiology, and biotechnology because of various biologically active compounds produced by the bacteria. The most pathogenic and well-studied species of the genus is *S. marcescens*. *S. marcescens* is a dangerous pathogen associated with nosocomial infections [37]. It causes respiratory and central nervous system diseases (meningitis), infections of urinary tract and skin, endocarditis, and sepsis [38]. Effective treatment of patients infected with *S. marcescens* is difficult, since this pathogen is resistant to most classes of antibiotics. *S. marcescens* is capable of secreting various enzymes into the environment. These include chitinase, lipase, chloroperoxidase, and protease [37,39]. In addition, the bacterium produces a natural red pigment, prodigiosin, possessing antifungal, antibacterial, and antiprotozoal activity [40]. Interestingly, when using bacterial extracts in the synthesis of silver nanoparticles, biomolecules and their fragments cover the surface of the particles and can impart additional functionalities to the particles [41,42]. Current data on silver nanoparticles synthesis using the genus Serratia are scarce and thus little is known about how these secreted substances participate in the particle synthesis and may confer different biological functions to the particles. AgNPs were earlier synthesized using *S. marcescens* MTCC8708 [41] and *S. nematodiphila* [43], but no comparison between nanoparticle synthesis using bacterial cellular filtrate and cell-free filtrate were made in these studies.

This study aims to conduct a comparative analysis of the involvement of intracellular and secreted extracellular compounds of *S. marcescens* in the synthesis of silver nanoparticles and to determine the inhibitory activity of the nanoparticles synthesized. The bacterial cellular filtrate was obtained by homogenizing the cell pellet using ultrasound followed by filtration. The cell-free filtrate was obtained by filtration of the bacterial suspension supernatant. Both bacterial filtrates yielded small (25–40 nm) spherical silver nanoparticles without the use of stabilizing chemicals. The silver nanoparticles demonstrated good antibacterial potential against the opportunistic bacterium *S. marcescens* and a streptomycin-resistant strain of *E. coli*.

It was found that *S. marcescens* cell-free filtrate inhibited growth of *E. coli*. Neither cellular nor cell-free filtrates exhibited inhibitory activity on growth of *S. marcescens*. Silver nanoparticles synthesized using *S. marcescens* filtrates did not have a high inhibitory effect on growth of *S. marcescens*.

The use of biologically synthesized silver nanoparticles in clinical settings will require further studies involving mammalian cell lines and a wide range of clinically relevant pathogens.

## 2. Results and Discussion

In this study, biogenic silver nanoparticles were synthesized using two types of bacterial filtrates, a cellular and cell-free filtrate. The color change in the solution during particle synthesis indicated the successful reduction reaction and the formation of silver nanoparticles [24,44,45]. Long synthesis times can be required for microbial nanoparticle production using cell-free supernatants [46] probably because of low concentration of reducing agents. A widely recognized mechanism of silver nanoparticle biosynthesis involves the enzyme-reduced nicotinamide adenine dinucleotide phosphate (NADPH)-nitrate reductase. Previously, such a synthesis mechanism was demonstrated using the fungus *Fusarium oxysporum* [31,47].

**Figure 1:**
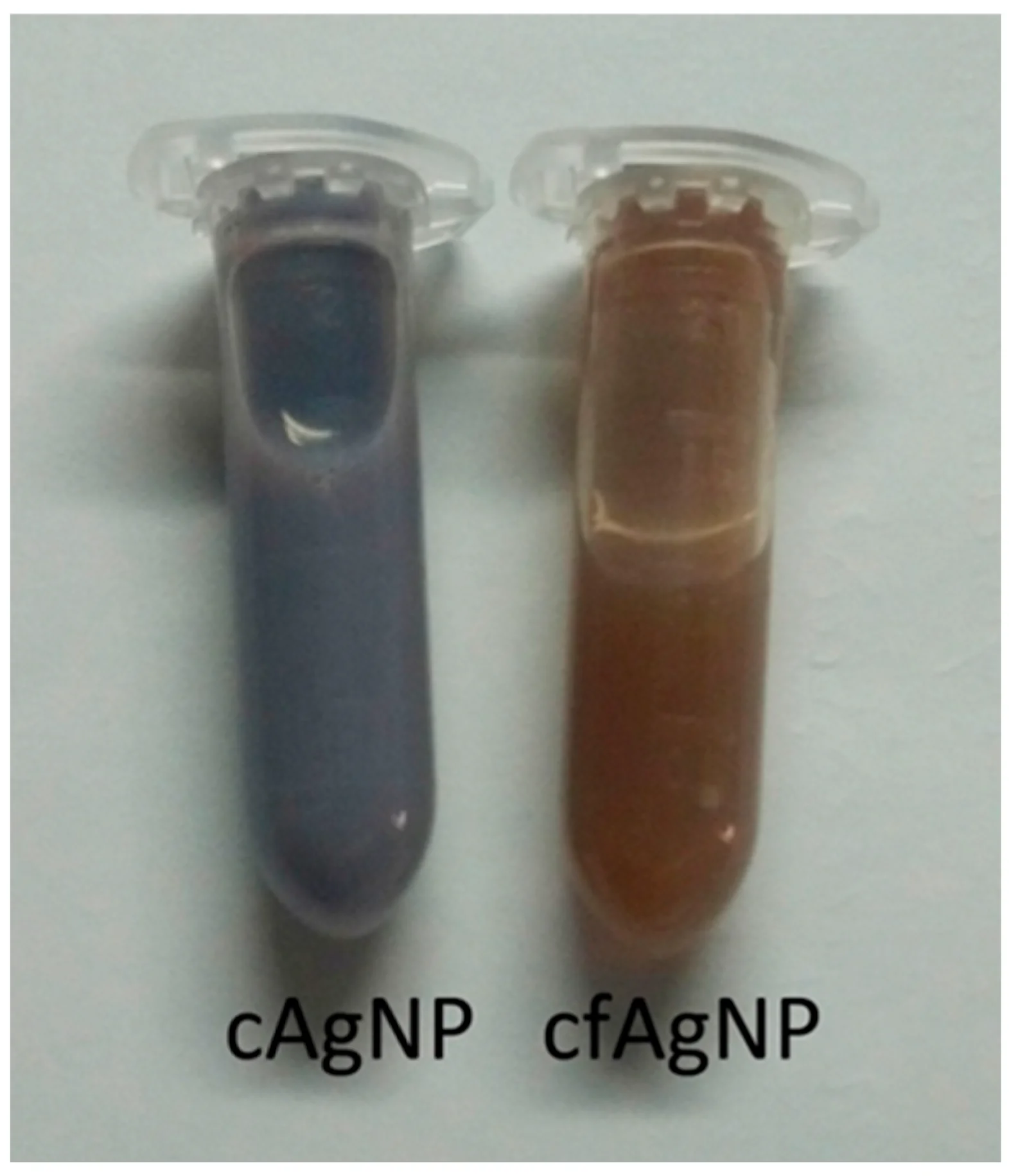
Suspensions of nanoparticles synthesized using different bacterial filtrates. cAgNPs are particles synthesized using cellular filtrate; cfAgNPs are particles synthesized using cell-free filtrate.

**Figure 2:**
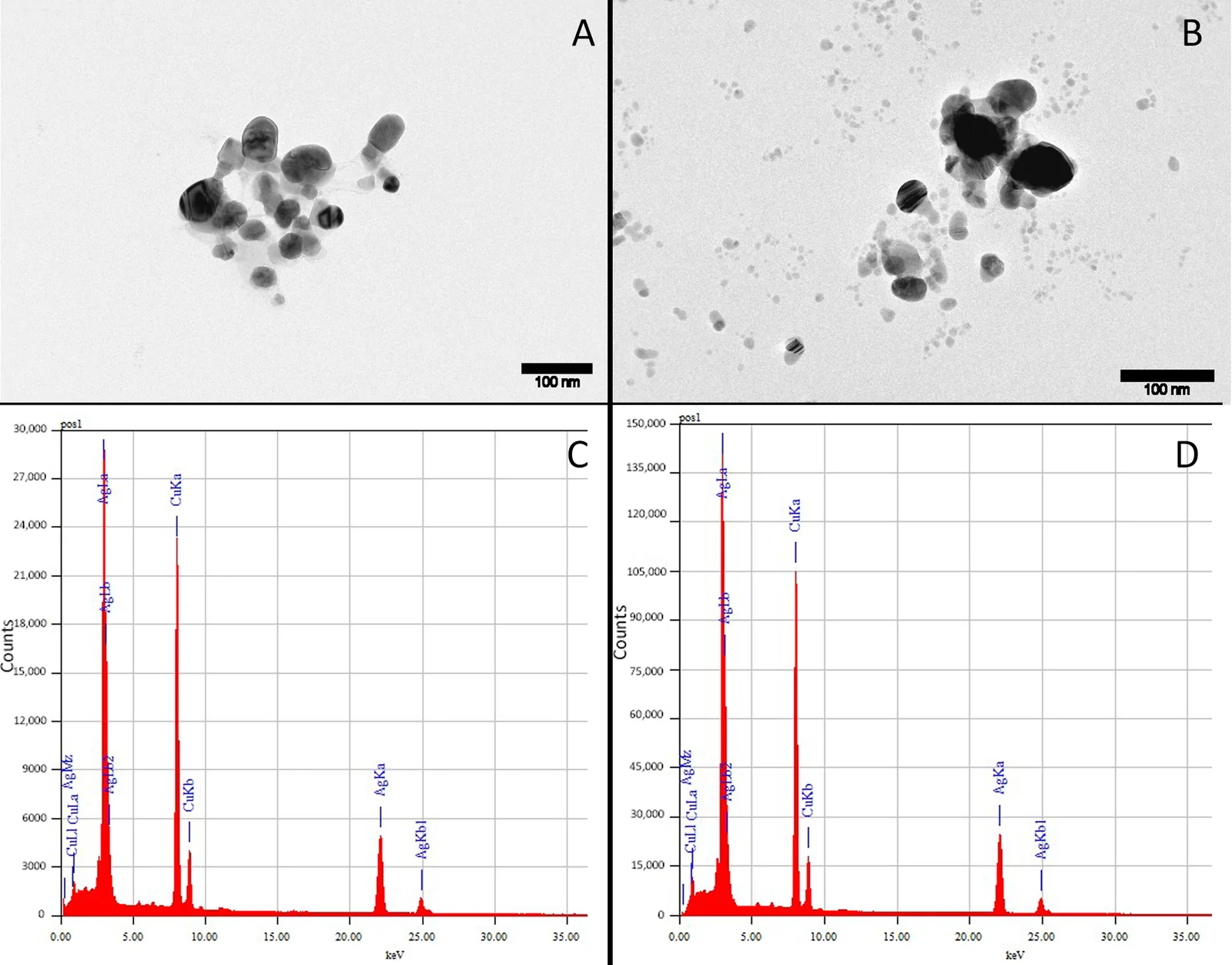
Transmission electron microscopy and energy-dispersive X-ray spectroscopy (EDX) of silver particles synthesized using cellular (**A**,**C**) and cell-free filtrate (**B**,**D**).

It is noteworthy that the color of the particle suspension synthesized using bacterial supernatant and cell filtrate differed. Suspension of particles synthesized using bacterial cell filtrate was a gray colour, while that synthesized using cell-free filtrate was brown (Figure 1), suggesting that the particles had different sizes. The different sizes of particles synthesized using *S. marcescens* filtrates were confirmed by hydrodynamic diameter measurements with Malvern Nano ZS analyzer. It was shown that silver nanoparticles stabilized by cell-free bacterial filtrate had a hydrodynamic diameter of 196.4 ± 6.2 nm, while silver nanoparticles stabilized by cell filtrate had a hydrodynamic diameter of 236.4 ± 1.3 nm. Characterization of the particle structure using transmission electron microscopy (TEM) demonstrated the spheroidal shape of the nanoparticles and sizes up to 40 nm (Figure 2). The particle size was 25.4 ± 10.6 nm for nanoparticles stabilized by cell-free bacterial filtrate, and 39.5 ± 12.1 nm for particles stabilized by cellular filtrate. The particle diameter measured by dynamic light scattering (DLS) is typically larger than the diameter measured by TEM [48]. In a liquid medium, a solvation shell forms around a dispersed particle, the thickness of which depends on various factors: for example, the structure of the stabilizing molecules. Also, such differences in particle size values can be explained by the aggregation of silver nanoparticles.

The particle size distribution in a solution is characterized by the polydispersity index (PdI), which is a measure of homogeneity in a dispersed system and can vary between 0 and 1. The polydispersity index is a numerical value for the particle size distribution. Homogeneity of particles in a sample is characterized by PdI values close to zero, while a PdI greater than 0.3 indicates heterogeneity of the sample. The polydispersity indexes of silver nanoparticles stabilized with bacterial filtrates indicate heterogeneous particle populations. The polydispersity index of the silver nanoparticles stabilized with cell-free filtrate was in the range of 0.375 ± 0.04. Particles synthesized using bacterial cell filtrate had a polydispersity index of 0.251 ± 0.02. The calculations used to measure the average hydrodynamic particle size and PdI are defined in ISO 22412:2017. Particles stabilized using bacterial filtrates had negative zeta potentials with different values, indicating the presence of different bacterial molecules on the particle surface (Table 1).

The composition of the nanoparticles was determined using energy-dispersive X-ray spectroscopy (EDX), which allows the analysis of the chemical composition of samples by measuring the energy of characteristic X-ray radiation. Figure 2C,D shows the energy-dispersive X-ray spectra of silver nanoparticles synthesized using two filtrates. Particle analysis revealed signals of silver atoms in all particles synthesized using bacterial filtrates. This signal is located in the 3 keV region due to surface plasmon resonance. The signals of O, C atoms (Figures S1 and S2) in bacterial filtrate samples can be due to X-ray emission from organic compounds of bacteria that participate in the synthesis of silver nanoparticles [49,50]. No peaks of silver atoms were detected in the spectra of bacterial filtrates.

**Figure 3:**
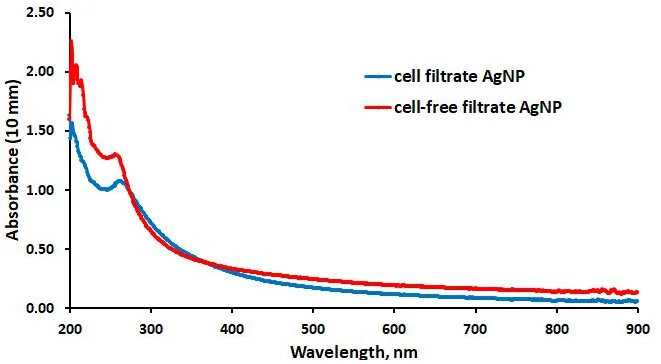
Absorption spectra of silver nanoparticles synthesized using cellular (**blue**) and cell-free (**red**) filtrates.

In UV–vis spectra, bacterial filtrates and nanoparticles had the same position of the absorption maximum (Figure 3 and Figure S3). Particles stabilized by cell-free filtrate had the absorption maximum at 257 nm, while those stabilized by cellular filtrate had the absorbance peak at 262 nm (Figure 3). The peak positions for particles stabilized by different filtrates varied slightly. The maximum absorption peaks of the synthesized silver nanoparticles at 257 nm and 262 nm are not typical for surface plasmon resonance peak of classical spherical silver nanoparticles (usually in the 400–450 nm range [18]). The peak positions of silver nanoparticles at about 250–260 nm were previously reported for some microbially synthesized nanoparticles and associated with silver–biomolecule complexes formed during biogenic synthesis [51]. Furthermore, the peak position varies proportionally to the size and shape of the nanoparticles and shifts toward longer wavelengths with increasing particle size [52]; larger particles also have a broader UV-visible absorption spectrum than smaller particles [53]. The absorption peak at around 260 nm can be explained by π→π * electron transition of the capping biomolecules and the Ag interband continuum, while a broad plateau around 400 nm corresponds to plasmonic resonance [54]. The close proximity of the peak maxima of particles stabilized by bacterial filtrates indicates that the particles are of approximately the same size, as confirmed by TEM (Figure 2) and DLS (Table 1).

We carried out spectrophotometric measurements of the nanoparticles after six months of storage as a dry powder, which revealed peaks at the same wavelengths (257 and 262 nm), demonstrating the stability of the synthesized silver nanoparticles (Figure S4). Thus, the nanoparticles can be safely stored as a dry powder and suspended in water or appropriate medium before application.

**Figure 4:**
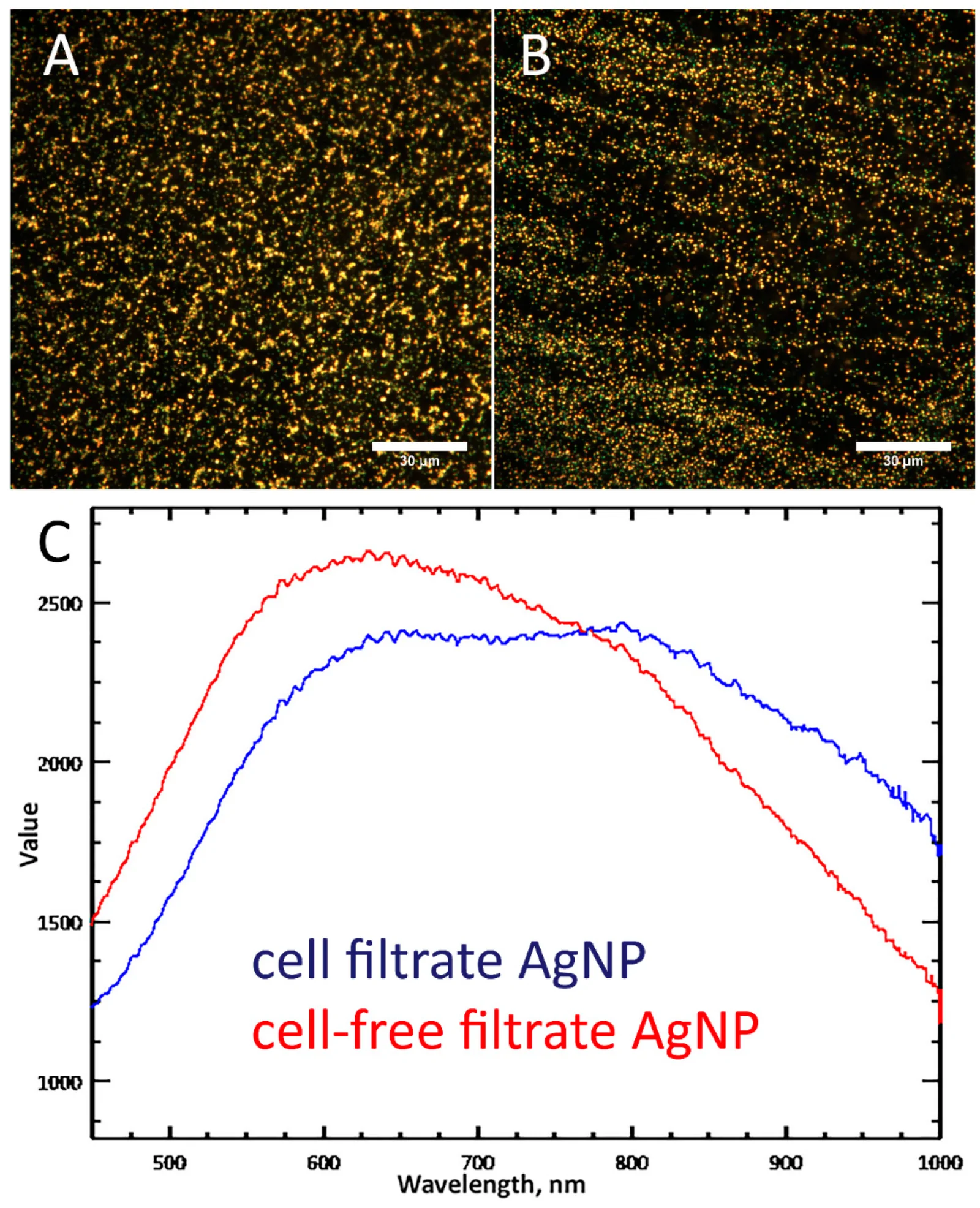
Dark-field microscopy of silver nanoparticles stabilized by cellular (**A**) and cell-free (**B**) filtrates and hyperspectral profiles of the particles (**C**).

Dark-field microscopy revealed that the particles had identical spectral properties (Figure 4A,B). The hyperspectral profiles of the bacterial filtrates were similar, without any distinct maxima. The spectra of the two particle types demonstrated a broad peak. The peak of the particles stabilized by cell-free filtrate was located in the visible spectrum (550–750 nm), in the yellow, orange, and red regions of the spectrum. The peak of particles stabilized by cellular filtrate was located in the 550–850 nm region, thus including infrared region of the spectrum in addition to the above three regions (Figure 4C).

**Figure 5:**
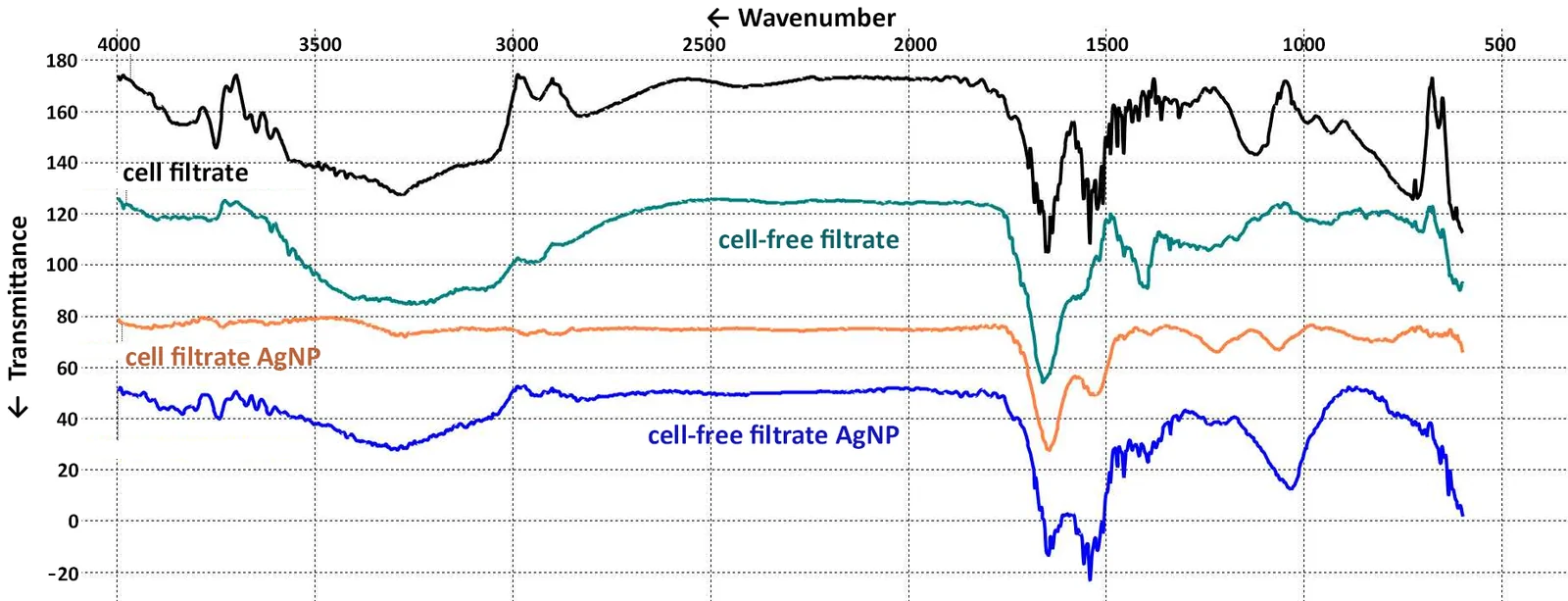
FT-IR spectra of cellular and cell-free filtrates of *S. marcescens* bacteria and filtrate-synthesized silver nanoparticles.

Bacterial and cell-free filtrates of microbial cultures are complex mixtures of many different substances and therefore have complex FTIR spectra (Figure 5). In the spectrum of the bacterial cell filtrate, a broad envelope band extending from 3700 to 2200 cm^−1^ represents the stretching vibration of the OH group. However, on this OH stretching peak, there is a narrower peak at 3289–3275 cm^−1^, which indicates the presence of N-H stretching vibrations in the spectrum. Small sharp peaks are visible at 3064 cm^−1^, 3079 cm^−1^, and 3099 cm^−1^, which may be peaks of CH stretching vibrations in an aromatic ring or another unsaturated compound. Small broad peaks near 2934 cm^−1^ and 2835 cm^−1^ contain asymmetric and symmetric CH stretching vibrations in methyl and methylene groups, as well as stretching vibrations of “single” hydrogen atoms in carbohydrates. The spectrum is dominated by two intense peaks at 1647 cm^−1^ and 1541 cm^−1^, which are typically found in the spectra of secondary amide groups. The first peak belongs to the stretching vibrations of the C=O group, and the second to the in-plane deformation vibration of N-H. The intense peak of the stretching vibration of the carbonyl group at 1647 cm^−1^ contains a number of smaller peaks, which may belong to C=O groups with different force constants (for example, in carboxylic acids), superimposed on the bands of carbonyls in secondary amides. Very narrow bands in the range of 1620–1400 cm^−1^ can probably be attributed to the stretching vibrations of the C-H aromatic ring. In addition, peaks of the stretching vibrations of C = C in alkenes can also be present in the region of 1680–1630 cm^−1^. The region of 1460–1300 cm^−1^ with numerous sharp peaks may include the bands of C-N stretching vibrations and CH deformation vibrations in methyl groups. The band at 1315 cm^−1^ may belong to C-O stretching vibrations in carboxylic acids. An intense and rather broad band at 1121 cm^−1^ with a shoulder at 1093 cm^−1^ probably belongs to a set of C-O stretching vibrations in carbohydrates. Small peaks in the region of 2000–1700 cm^−1^ are probably overtones and combined bands of the aromatic ring. A broad band near 932 cm^−1^ may include a peak of out-of-plane OH deformation vibrations in carboxylic acids. Out-of-plane deformation vibrations of the NH_2_ amino group are usually observed in the region of 850–750 cm^−1^. The broad band at approximately 725 cm^−1^ is likely responsible for out-of-plane N-H and O-H bending vibrations, respectively. The sharp peak at 715 cm^−1^, adjacent to the peak at 725 cm^−1^, may be due to out-of-plane C-H bending vibrations in the aromatic ring. Thus, according to the IR spectra, the bacterial cell filtrate likely contained proteins or peptides, carbohydrates, carboxylic acids, and compounds with saturated and unsaturated C-C bonds. It should be noted that the amino acid residues in peptides can also be diverse and include aromatic, amine, hydroxyl, and carboxyl groups.

In the spectrum of the cell-free filtrate of the bacterial culture, a broad and intense band from 3696 cm^−1^ to 2564 cm^−1^ likely arises from O-H stretching vibrations, but N-H stretching vibrations may also be present in this region. The bands at 2948 cm^−1^ and 2872 cm^−1^, located to the right of this massive peak, represent CH stretching vibrations in the methylene and methyl groups. There are also several small peaks in the OH stretching band, which may belong to CH sites in unsaturated compounds. The spectrum is dominated by a peak at 1660 cm^−1^ (with a double-peaked shoulder (1576 and 1567 cm^−1^)), belonging to carbonyl (C=O) stretching vibrations. The peak is broad at the base and spans the wavenumber range from 1758 cm^−1^ to 1484 cm^−1^, and its shape is strongly distorted, as if at least one additional peak were superimposed on its right side. Moreover, the width of this peak suggests that it may span multiple C=O peaks with different force constants. The second most intense peak in the spectrum is located at 1405 cm^−1^. We propose that this peak belongs to the symmetric stretching vibrations of C–O bonds in carboxylates, while the accompanying peak of asymmetric stretching vibrations of C–O, which typically lies in the range of 1650–1540 cm^−1^, is superimposed on the right side of the C=O peak at 1660 cm^−1^. The band of the in-plane deformation vibration of N-H in the secondary amide group, hidden in the large C=O peak, may also be located here, since the band of the in-plane deformation vibration of N-H in amides usually lies between 1570 cm^−1^ and 1515 cm^−1^. A broad band is also observed between 1367 cm^−1^ and 1050 cm^−1^, centered at approximately 1252 cm^−1^. In this region, the C-O stretching vibrations in carboxylic acids, the C-C-O stretching vibrations in ethers and alcohols, the C-C-C stretching vibrations in ketones, and the in-plane deformation vibrations of OH in alcohols may occur. Two broad bands are also observed at 1040–850 cm^−1^ and 790–700 cm^−1^. These regions typically contain C-C-O stretching vibrations in alcohols, symmetric C-O-C stretching vibrations in esters, out-of-plane OH bending vibrations, and out-of-plane N-H bending vibrations in amides.

In the spectrum of AgNPs synthesized using cell filtrate, two prominent peaks are observed at 1650 cm^−1^ and 1536 cm^−1^ (Figure 5). These peaks can be attributed to C=O stretching vibrations and NH in-plane bending vibrations, indicating the presence of secondary amide groups. These peaks were also the most intense in the spectrum of cell filtrate. The region between 3367–3152 cm^−1^ contains N-H stretching vibrations, while the peak at 1393 cm^−1^ may be due to C-N stretching vibrations. Two small broad peaks near 2963 cm^−1^ and 2880 cm^−1^ are attributed to C-H stretching vibrations in the methyl and methylene groups. There are also two broad peaks near 1222 cm^−1^ and 1064 cm^−1^. C-O stretching bands are usually observed in these regions. Analysis of the spectra of AgNPs prepared using the cell-free filtrate of the microbial culture confirms our earlier hypothesis that the cell-free filtrate spectrum contained a peak of N-H in-plane vibrations in amide groups hidden in a large neighboring C=O peak, since this N-H in-plane vibration peak is clearly visible in the spectrum of AgNPs synthesized using the cell-free filtrate. The spectra of both AgNPs are very similar to each other, as well as to the spectra of the cellular and cell-free filtrates. Most of the peaks in the 3600–1300 cm^−1^ region (including very small bands) retain their positions in all four spectra. However, in the spectra of the cellular and cell-free filtrates, broad peaks of C-O stretching vibrations were located around 1121 cm^−1^ and 1242 cm^−1^, respectively, and intense bands between 1030 cm^−1^ and 1060 cm^−1^ were absent. In the spectra of AgNPs, instead of these C-O peaks, an intense C-O stretching peak appeared at 1033 cm^−1^ in AgNPs stabilized with extracellular filtrate and at 1064 cm^−1^ in AgNPs stabilized with cellular filtrate. A decrease in the intensity of the 3700–3000 cm^−1^ band was also observed for both AgNPs. An increase in the intensity of the C-O stretching peak, a decrease in the intensity of the O-H stretching band, and a change in the position of some peaks were previously observed in the spectrum of the biopolymer chitosan after its interaction with AgNPs [55]. Moreover, in another study, a positional shift could also be seen in the spectrum of AgNPs synthesized using a plant extract, along with a significant increase in the intensity of the C-O stretching band [56]. Thus, in our work, the presence of proteins, peptides, or other substances containing secondary amide groups is noticeable in the spectra of both AgNPs. The increase in the intensity of the C–O peak and the decrease in the intensity of the O–H stretching peak suggest that the oxygen atoms in the C–O bonds were likely involved in the interaction with the AgNPs and therefore were less involved in the formation of hydrogen bonds.

Although FT-IR data confirm the presence of organic moieties on the nanoparticles, it should be underlined that FT-IR data cannot distinguish between adsorption and co-modification, nor can they confirm that these molecules mediate strain-specific effects.

**Figure 6:**
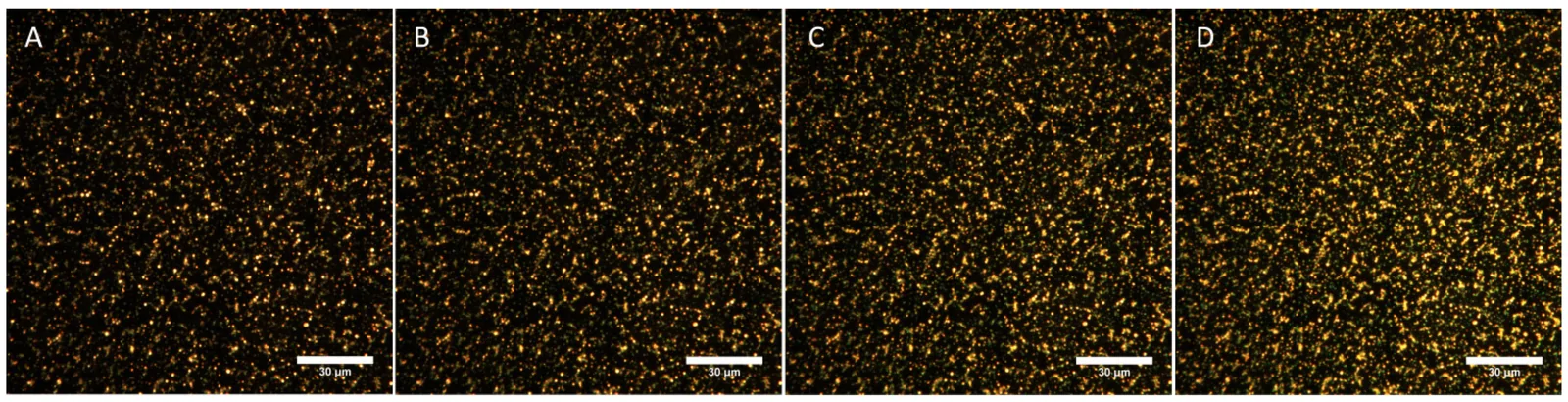
Reduction of silver ions associated with silver nanoparticles synthesized using cell filtrate under exposure to visible blue light for 0 (**A**), 2 (**B**), 5 (**C**), and 10 (**D**) minutes.

**Figure 7:**
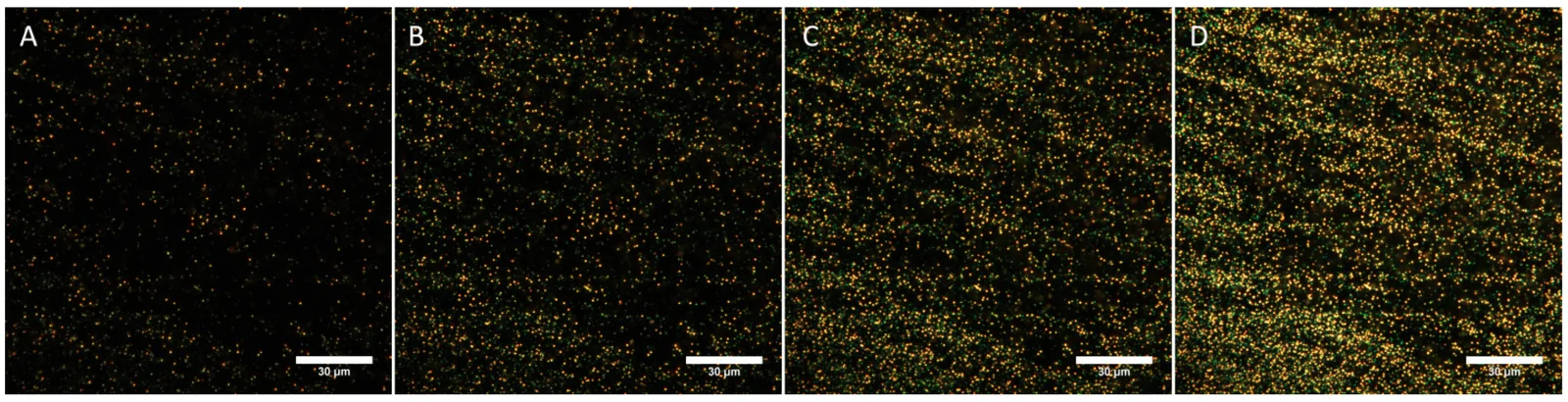
Reduction of silver ions associated with silver nanoparticles synthesized using cell-free filtrate under exposure to visible blue light for 0 (**A**), 2 (**B**), 5 (**C**), and 10 (**D**) minutes.

Similar to the results obtained in our earlier work, we noted the further reduction of silver ions associated with silver nanoparticles during exposure to blue light [18]. Figure 6 and Figure 7 show dark-field photographs of filtrate-stabilized silver nanoparticles taken over different time intervals—0, 2, 5, and 10 min—demonstrating the change in the spectral properties of the particles over time. This phenomenon confirms that both bacterial filtrates contain blue light-sensitive agents, for example, proteins or peptides containing tyrosine, which are involved in the reduction of silver ions [57,58].

**Figure 8:**
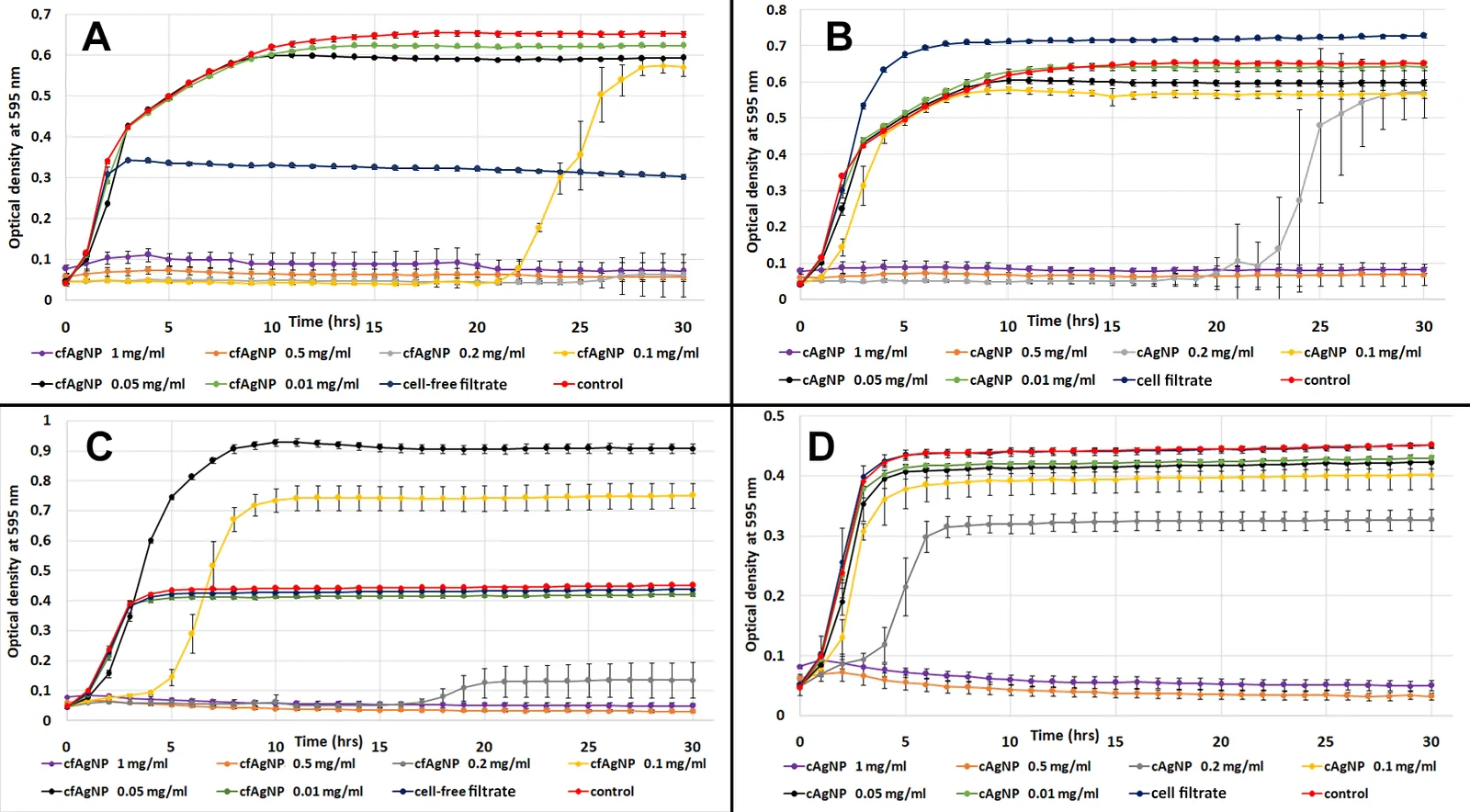
Growth dynamics of bacterial cultures in the presence of filtrate-stabilized silver nanoparticles. (**A**,**B**)—*E. coli*; (**C**,**D**)—*S. marcescens* (*n* *=* 3–7).

**Figure 9:**
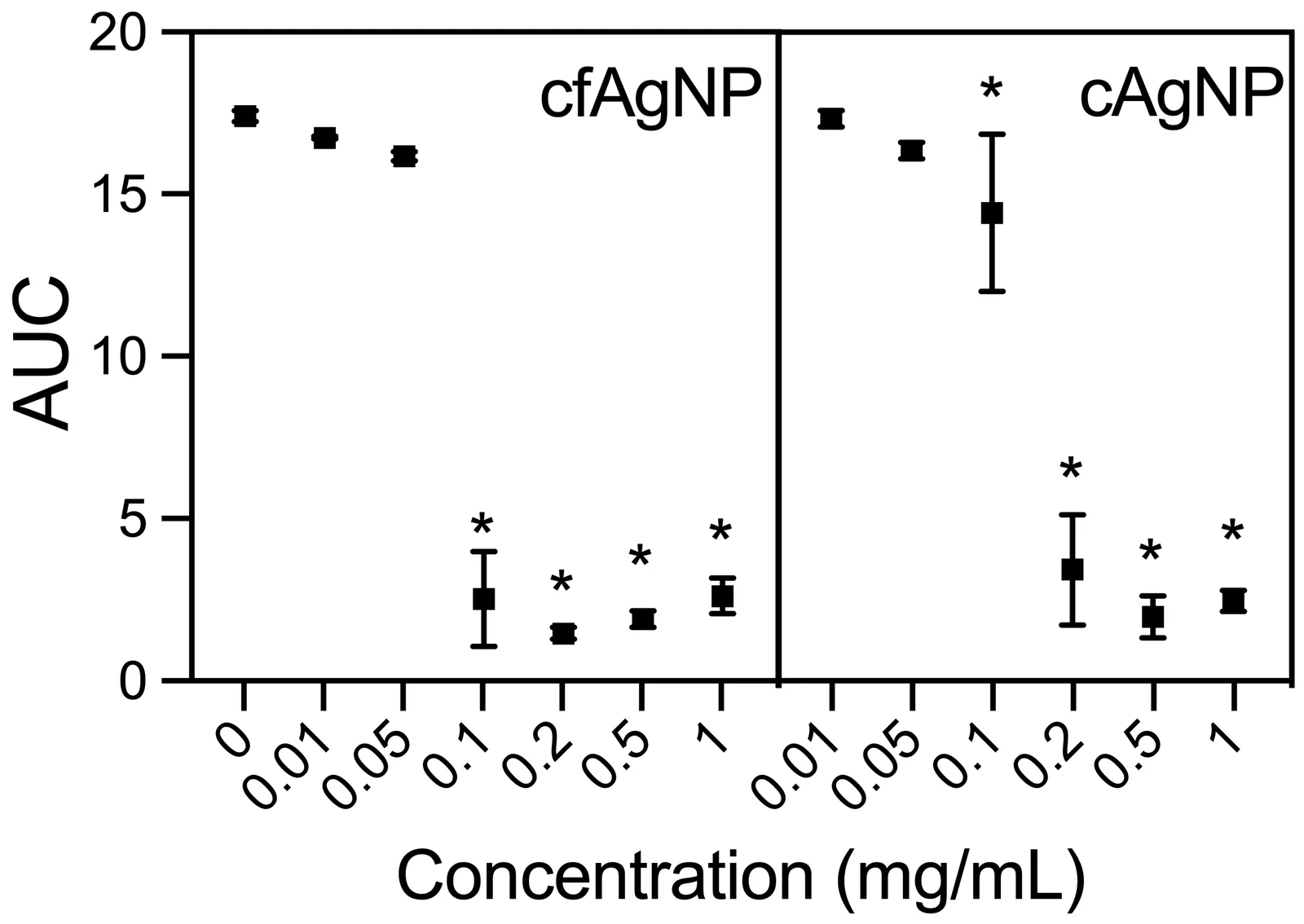
Effect of cfAgNP and cAgNP on total growth of *E. coli* OP-50, expressed as area under the growth curve (AUC). Cultures were exposed to particles at 0.01–1 mg/mL and growth was monitored by OD595 over 30 h. Data presented as mean ± SD. One-way ANOVA followed by Dunnett’s post hoc test was performed to assess differences from control group (* *p* < 0.05).

**Figure 10:**
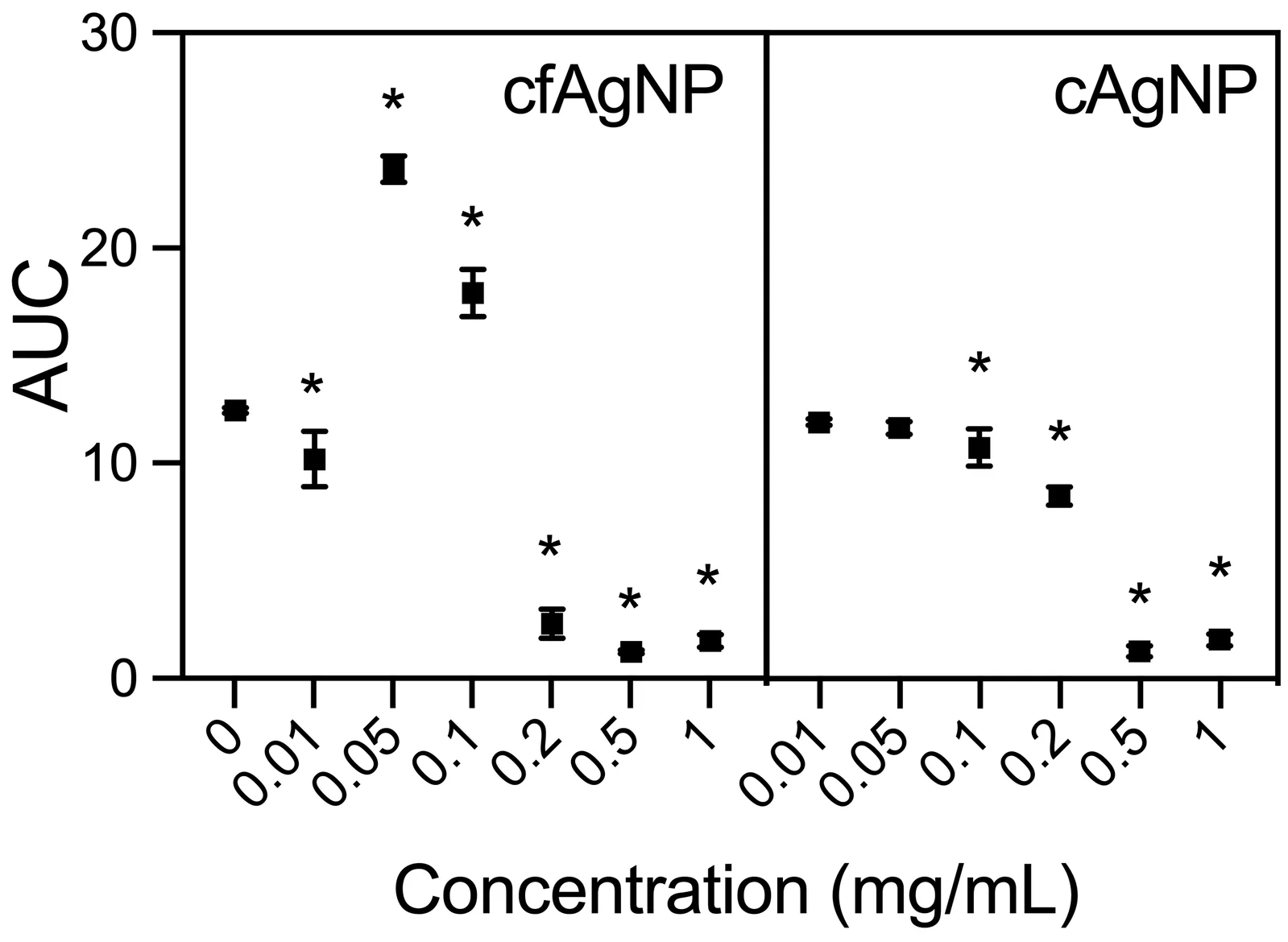
Effect of cfAgNP and cAgNP on total growth of *S. marcescens*, expressed as area under the growth curve (AUC). Cultures were exposed to particles at 0.01–1 mg/mL and growth was monitored by OD595 over 30 h. Data presented as mean ± SD. One-way ANOVA followed by Dunnett’s post hoc test was performed to assess differences from control group (* *p* < 0.05).

Silver nanoparticles synthesized using two different bacterial filtrates exerted different effects on the growth and reproduction of *E. coli* and *S. marcescens* (Figure 8, Figure 9 and Figure 10).

Bacterial growth was monitored by optical density at 595 nm (OD595) at hourly intervals over 30 h, with 3–7 replicates per group. For each well, the area under the growth curve (AUC) was calculated as an integrated measure of total growth. For streptomycin-resistant *E. coli*, cfAgNPs reduced bacterial growth at concentrations 1, 0.5, 0.2, and 0.1 mg/mL, with AUC values of 2.61 ± 0.55, 1.90 ± 0.25, 1.46 ± 0.18, and 2.52 ± 1.46, respectively, all significantly lower than the untreated control (17.40 ± 0.17; *p* < 0.05). At concentrations of cfAgNPs of 0.05 and 0.01 mg/mL, growth did not differ from the control (16.16 ± 0.14 and 16.74 ± 0.04). The cAgNPs fraction showed the same pattern, with significant growth reduction at 0.1–1 mg/mL (AUC 2.47 ± 0.31, 1.99 ± 0.64, 3.43 ± 1.71, and 14.44 ± 2.42 at 1, 0.5, 0.2, and 0.1 mg/mL, respectively) and no significant effect at 0.05 and 0.01 mg/mL (16.36 ± 0.25 and 17.34 ± 0.25). The cell-free filtrate showed reduced growth relative to the untreated control (AUC 8.87 ± 0.84; *p* < 0.05), whereas the cell filtrate showed increased growth with AUC 19.86 ± 0.12 (*p* < 0.05) (Figure 9). MIC of cell-free AgNPs for *E. coli* was 0.1 mg/mL. MIC of cell AgNPs for *E. coli* was 0.2 mg/mL.

For opportunistic bacteria *S. marcescens*, cfAgNPs significantly reduced growth at 1, 0.5 and 0.2 mg/mL (AUC 1.76 ± 0.29, 1.23 ± 0.09 and 2.56 ± 0.67, respectively) compared to control (12.46 ± 0.13; *p* < 0.05). At the concentrations of 0.1 and 0.05 mg/mL, AUC values exceeded the control (17.93 ± 1.10 and 23.67 ± 0.62; *p* < 0.05), while at 0.01 mg/mL, growth was slightly reduced to 10.18 ± 1.29. cAgNPs reduced growth at 0.1–1 mg/mL (AUC 1.82 ± 0.27, 1.30 ± 0.25, 8.49 ± 0.41, and 10.74 ± 0.86 at 1, 0.5, 0.2, and 0.1 mg/mL, respectively; *p* < 0.05), with no effect at 0.05 and 0.01 mg/mL. Neither the cell-free filtrate nor the cell filtrate differed from the control (12.12 ± 0.05 and 12.51 ± 0.27) (Figure 10).

The higher concentrations of silver nanoparticles required for the antibacterial activity in our study compared to other studies can be explained by differences in the size and charge of the particles used in different studies. It has been shown that the larger the particle size, the lower the antibacterial activity of nanoparticles [16]. Furthermore, negatively charged particles (as in our study) are less effective than positively charged ones [16], as negatively charged particles interact less with the cell wall. This study only compared the in vitro activity of silver nanoparticles synthesized using cellular and cell-free filtrates against two Gram-negative bacterial strains. The biomedical application of the nanoparticles will require additional studies using clinically relevant Gram-positive and Gram-negative pathogens and cytotoxicity assessment on mammalian cells.

Interestingly, the cell-free filtrate exhibited an inhibitory effect on *E. coli*, while the cell filtrate did not. Furthermore, the study did not demonstrate that filtrates containing compounds obtained from *S. marcescens* exhibited high effective activity against *S. marcescens* itself, the source of the isolate. Conversely, the cell-free filtrate was shown to be effective against the opportunistic Enterobacteriaceae *E. coli*. The possible presence of organic compounds with antimicrobial activity in the bacterial cell-free filtrate of *S. marcescens* leading to the presence of these compounds on the particle surface make this mixture ideal for producing antibacterial agents. We attributed differences in activity to bacterial biomolecules adsorbed on the nanoparticle surface because it is the most plausible explanation given the nonspecific antibacterial action of the nanoparticle silver core. The primary differences in nanoparticle capping biomolecules can influence other nanoparticle properties such as size, zeta-potential and aggregation which are also important for nanoparticle efficiency. However, the mechanism remains speculative because it was not experimentally tested.

## 3. Materials and Methods

### 3.1. Cell Filtrate Preparation

*S. marcescens* bacteria ATCC 9986 were cultured in glucose-peptone medium at 27 °C. The cell lysate was prepared using a modified method described previously [35]. Forty ml of the overnight *S. marcescens* bacterial culture was centrifuged at 12,000 rpm for 10 min, and the pellet was washed with sterile phosphate-buffered saline and resuspended. Bacteria were lysed using an ultrasonic disperser (Bandelin Sonopuls HD 2070, Bandelin, Berlin, Germany) at 4 °C for 40 min. After the treatment, the cells were centrifuged at 13,000 rpm for 1 h. The supernatant was filtered using a syringe filter (Corning^®^ 28 mm Diameter Syringe Filters, 0.2 µm Pore PES Membrane, Corning Inc., Corning, NY, USA) with a pore size of 0.2 µm and stored at 4 °C.

### 3.2. Preparation of Cell-Free Filtrate

Preparation of cell-free filtrate was performed as previously described by Allam et al. (2019) [30]. Forty ml of the overnight bacterial culture was pelleted at 6000 rpm for 10 min. The precipitate was mixed with 15 mL of distilled water, incubated for 24 h, and precipitated at 13,000 rpm for 1 h, and the supernatant was filtered using a syringe filter (Corning^®^ 28 mm Diameter Syringe Filters, 0.2 µm Pore PES Membrane, Corning Inc., Corning, NY, USA) with a pore size of 0.2 µm. The supernatant was stored at 4 °C.

### 3.3. Synthesis of Silver Nanoparticles

**Figure 11:**
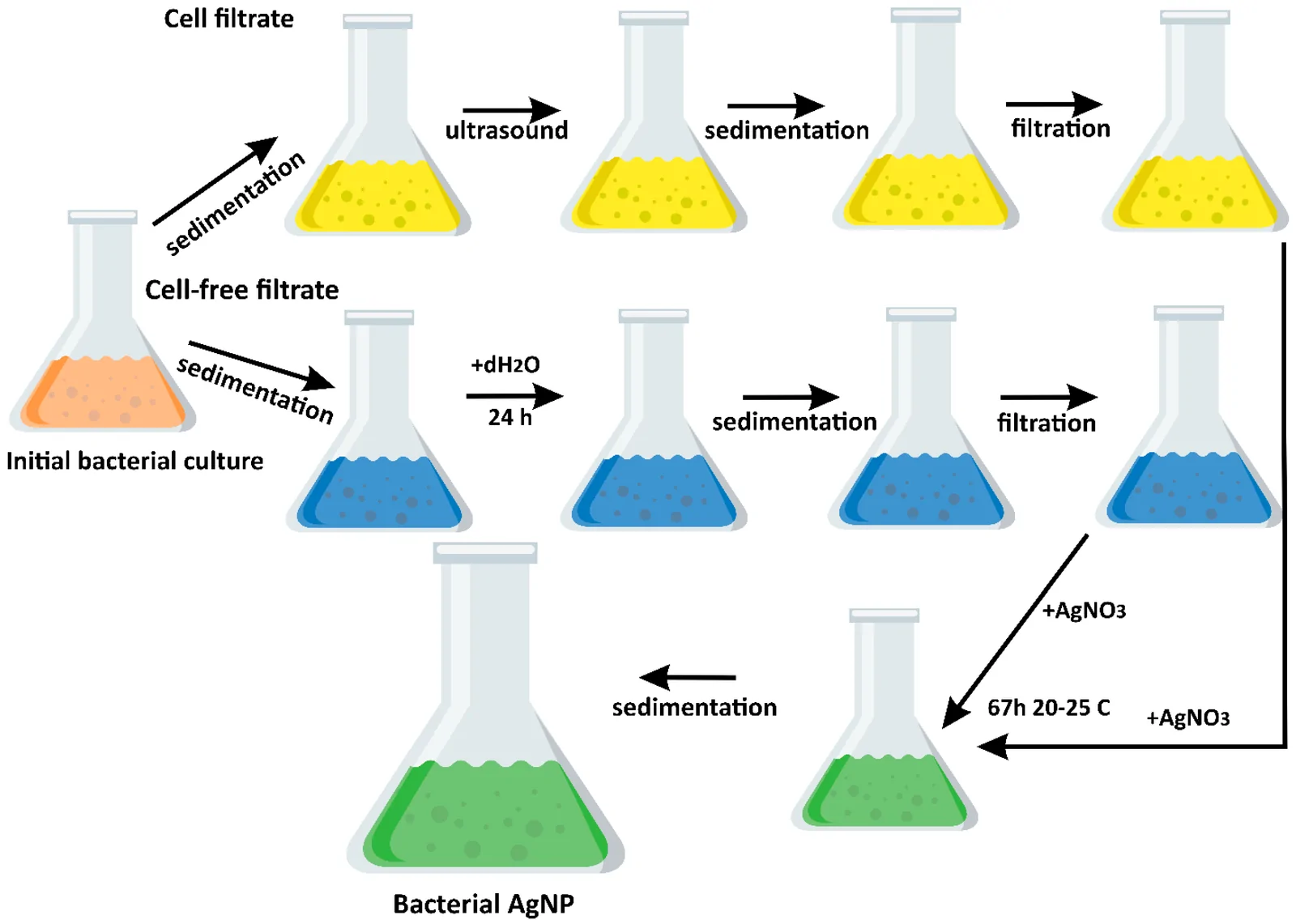
Schematic representation of the production of silver nanoparticles using cellular and cell-free filtrates.

Nine ml of 1 mM AgNO_3_ was added to 10 mL of each filtrate. All works were performed under sterile conditions using sterile distilled water to prevent the introduction of various impurities. The mixture was incubated with stirring at room temperature (20–25 °C) for 67 h. Relatively long synthesis time (67 h) was used to ensure high levels of silver conversion to nanoparticles. The period of 24 h was also insufficient for microbial AgNPs synthesis in other studies that involved cell-free supernatants [46], probably because of low concentration of reducing agents. The synthesis of nanoparticles was monitored by measuring the optical density of the sample solutions and observing color changes, but silver conversion yield was not quantitatively determined. The precipitate was then centrifuged at 13,000 rpm for 10 min, and the particle precipitate was washed with distilled water. The particles were dried at 40–45 °C. A schematic process of the particle synthesis from the preparation of bacterial filtrates to the production of silver nanoparticles is shown in Figure 11.

### 3.4. Particle Size and Zeta Potential

The hydrodynamic diameter and zeta potential of the nanoparticles were determined using a Zetasizer Nano ZS instrument (Malvern Panalytical Ltd., Malvern, UK) with DTS1070/DTS1080 disposable capillary cuvettes at 25 °C. One mL (0.125 mg/mL) of a sample was placed into disposable capillary cuvette.

### 3.5. Spectrophotometric Measurements

Spectrophotometric registration of the particles was performed in 10 mm quartz cuvettes using a NanoPhotometer NP80 spectrophotometer (Implen, München, Germany). One mL (0.125 mg/mL) of a sample was placed into quartz cuvettes.

### 3.6. Fourier Transform Infrared Spectroscopy

Infrared spectroscopy of the samples was performed on a FT-801 FTIR spectrophotometer equipped with an ATR accessory with a Zn-Se element (Simex, Novosibirsk, Russia). Spectra were recorded in the range from 600 to 4000 cm^−1^ at a resolution of 4 cm^−1^ and averaged over 26 scans. Scans were processed using the Zair 3.5 software (Simex, Novosibirsk, Russia). Dry powder samples were analyzed using a FT-801 FTIR spectrophotometer.

### 3.7. Transmission Electron Microscopy and Energy-Dispersive X-Ray Spectroscopy (EDX)

Structure and morphology of the nanoparticles was studied using a JEM-2100 transmission electron microscope (TEM) (JEOL, Tokyo, Japan) with a magnification of 50–1 500,000 times and an image resolution of 0.19 nm at an accelerating voltage of 200 kV.

Samples (10 µL) were dispersed in distilled water (0.5 mL). The resulting solution was applied to a copper grid. The sample holder was cleaned in a plasma chamber to avoid the ingression of organic compounds into the microscope. The average particle size of nanoparticles was calculated using the Image J software V 1.8.0.

Scanning transmission electron microscopy (STEM) was performed in combination with energy-dispersive X-ray spectroscopy (EDX) to determine the elemental distribution in the samples using a JEM-2100 instrument (JEOL, Tokyo, Japan). Sample preparation was carried out similarly for transmission electron microscopy.

### 3.8. Darkfield Microscopy and Hyperspectral Evaluation

Darkfield images and reflected light spectra were acquired using an Olympus BX51 upright microscope (Olympus, Tokyo, Japan) equipped with a CytoViva^®^ enhanced darkfield condenser (CytoViva, Auburn, Al, USA) and a Fibre-Lite DC-950 halogen light source (150 W) (Dolan Jenner Industries Inc., Boxborough, MA, USA). Water suspensions of nanoparticles were dropped onto clean glass slides and covered by cover slips. Darkfield images were acquired at the exposure times of 125–500 ms using Exponent 7 software (Dage-MTI, Michigan City, IN, USA). Spectra were recorded using a Specim V10E spectrograph (Spectral Imaging LTD, Oulu, Finland) and a Pixelfy.usb CCD camera in the wavelength range of 450–1000 nm with a spectral resolution of 2 nm. Hyperspectral image (HSI) data were collected with an exposure time of 0.25–0.6 s and full illumination intensity, which took approximately 11–30 min to obtain a full scan of 900 lines. HSI data were stored and analyzed as hypercubes using ENVI v. 4.8 software (Harris Geospatial Solutions, Broomfield, CO, USA) [59,60]. The data were processed using the software’s internal correction algorithm to remove lamp spectra. Averaged hyperspectral signals from 250–260 individual particles of all colors (10,000 pixels) were obtained. Hyperspectral libraries were created for all silver particles.

### 3.9. Growth Dynamics of S. marcescens and Streptomycin-Resistant E. coli OP-50

Cell growth in the presence of particles was assessed in a 96-well microplate (Corning Inc., Corning, NY, USA). Growth medium (200 µl), 10 µl of cell suspension (OD 1 o.u.), and 10 µl of nanoparticles/filtrates at different concentrations (0.01; 0.05; 0.1; 0.2; 0.5, and 1 mg/mL) were added to each well. The microplate was covered with optically transparent film and incubated at 27 °C and 37 °C for *S. marcescens* and *E. coli*, respectively. Absorbance was recorded at 595 nm using a microplate reader (Thermo Scientific Multiscan FC, Thermo Fisher Scientific Inc., Waltham, MA, USA) for 30 h. The results were processed using Microsoft Excel.

### 3.10. Statistical Data Analysis

All experiments were performed in triplicate, and the reported results represent the mean ± standard error. Statistical analysis of growth dynamics was conducted using analysis of area under curve, AUC. For each well, the area under the growth curve was calculated over the entire 30 h period. AUC is an integral measure of overall growth that combines the lag phase, growth rate, and final density into a single number [61]. AUC values are reported as mean ± standard deviation. Prior to comparison, the normality of each group’s distribution was assessed using the Shapiro–Wilk test. Since most data met the assumption of normality, AUC values were compared relative to the control using one-way ANOVA with Dunnett’s post hoc test. A *p*-value < 0.05 was considered statistically significant.

## 4. Conclusions

Green synthesis using bacterial compounds is a promising and environmentally friendly approach to producing silver nanoparticles. Bacterial lysates are complex mixtures of diverse compounds with varying biological activity [62]. These mixtures yield silver nanoparticles of varying sizes, shapes, and activities [23,63].

In this study, we analyzed the influence of components of two types of bacterial filtrates on the synthesis of silver nanoparticles and compared the properties of resulting silver nanoparticles. We demonstrated that the use of either *S. marcescens* bacterial filtrate resulted in the synthesis of small, spherical silver nanoparticles (25.4 ± 10.6 nm for cfANPs and 39.5 ± 12.1 nm for cAgNPs) with similar spectral properties.

Nanoparticles cfAgNPs at 1, 0.5, 0.2 and 0.1 mg/mL and cAgNPs at 1–0.1 mg/mL demonstrated high growth-inhibitory activity against *E. coli* under the present experimental conditions.

It was shown that nanoparticles with “native” compounds did not have higher inhibitory activity against the bacterial source of the filtrates. This study demonstrated that under the present experimental conditions, the bacterial cell-free filtrate of *S. marcescens* had inhibitory activity against *E. coli*, but not against *S. marcescens*.

## Figures and Tables

**Table 1:** Hydrodynamic diameters, zeta-potentials, and polydispersity indices of silver nanoparticles (*n* = 3).

Sample	Hydrodynamic Diameter, nm	Zeta-Potential, mV	Polydispersity Index
Silver nanoparticles stabilized by bacterial cell filtrate (cAgNPs)	236.4 ± 1.3	−26.6 ± 0.8	0.251 ± 0.02
Silver nanoparticles stabilized by bacterial cell-free filtrate (cfAgNPs)	196.4 ± 6.2	−38.8 ± 0.6	0.375 ± 0.04

## Data Availability

The original contributions presented in this study are included in the article/Supplementary Material. Further inquiries can be directed to the corresponding author.

## Supplementary Materials

The following supporting information can be downloaded at: https://www.mdpi.com/article/10.3390/ijms27167150/s1.
