# Supplementary material for: Extracellular and Intracellular Bacterial Compounds in the Synthesis of Inorganic Particles: Silver Nanoparticles
Source: Int J Mol Sci. 2026 Aug 10;27(16):7150. doi: 10.3390/ijms27167150 (PMC13513195; doi:10.3390/ijms27167150)
Supplement: Supplementary file 1 [file ijms-27-07150-s001.zip › ijms-4324636-supplementary.pdf]

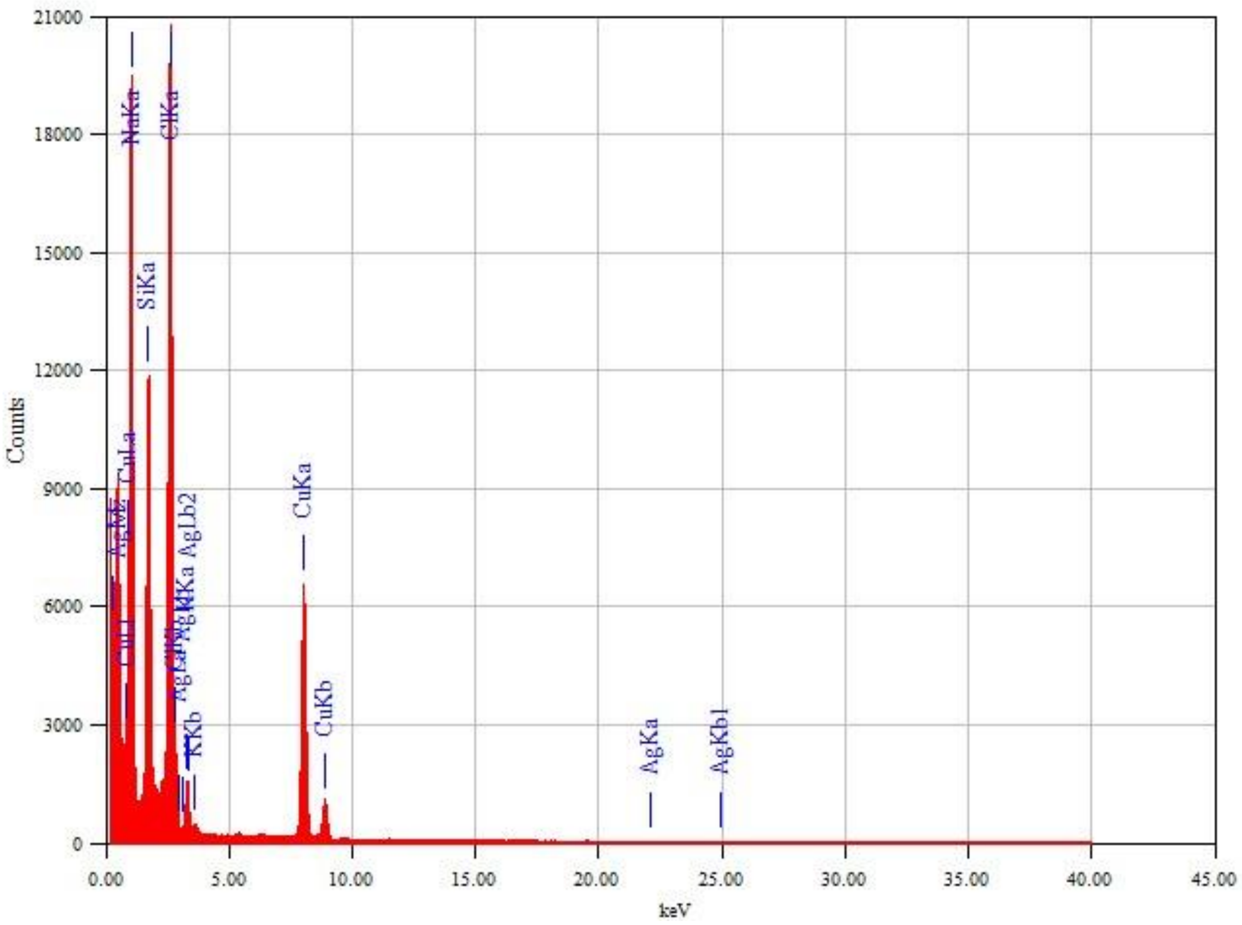

Figure S1. Energy-dispersive X-ray spectroscopy of cell-free filtrate.

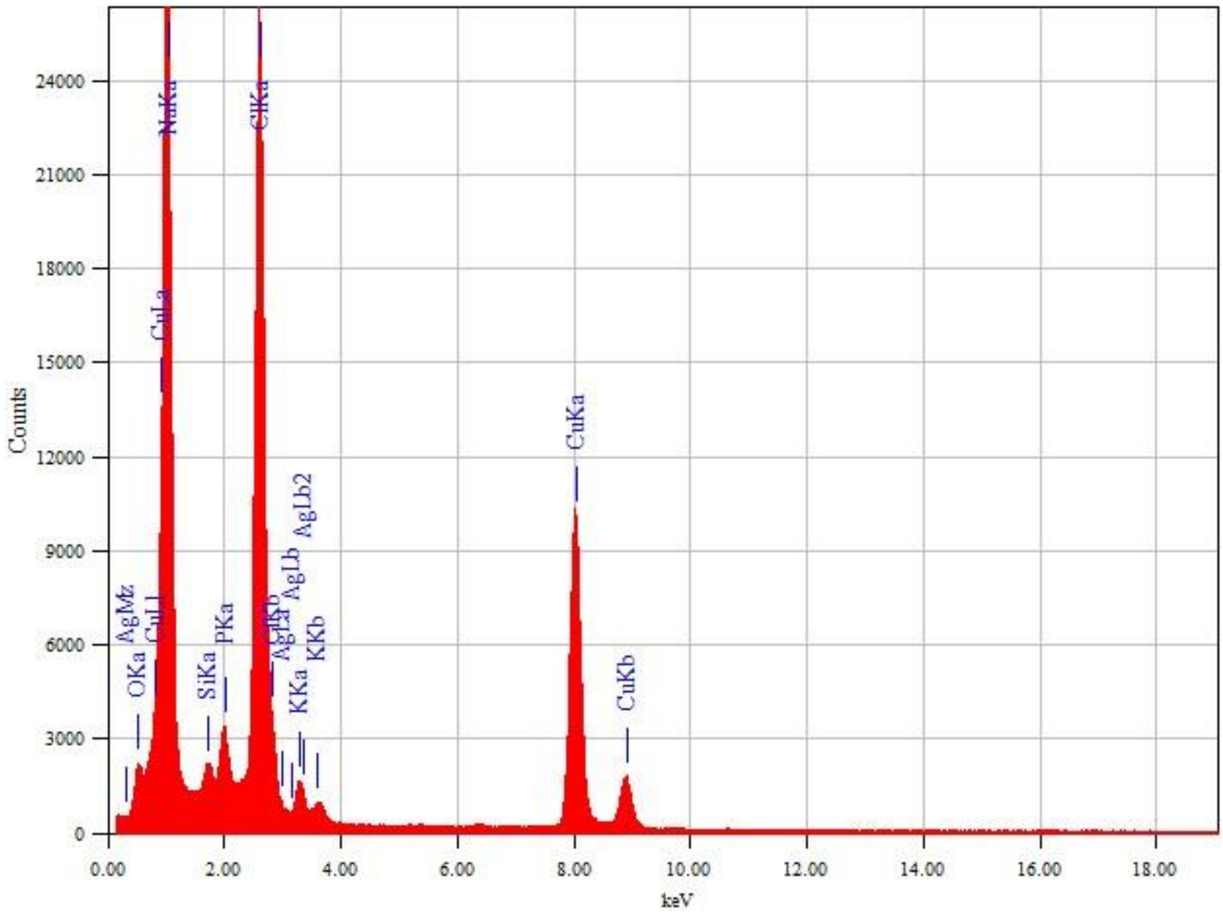

Figure S2. Energy-dispersive X-ray spectroscopy of cellular filtrate.

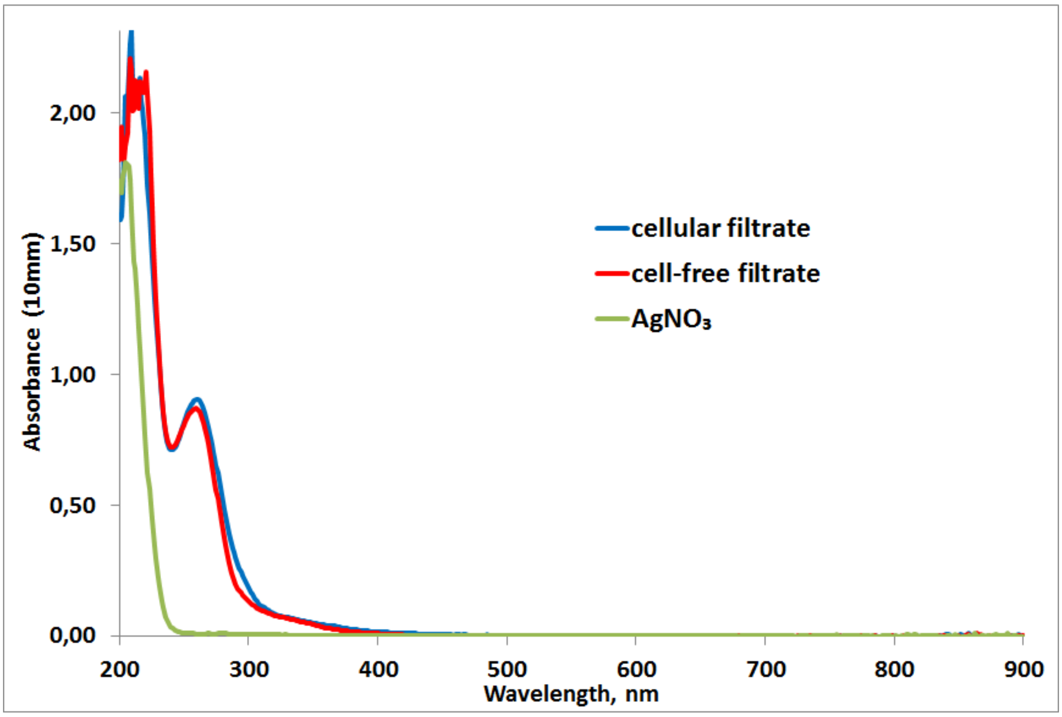

Figure S3. Absorption spectra of cellular (blue), cell-free (red) filtrates and $AgNO_3$ (green).

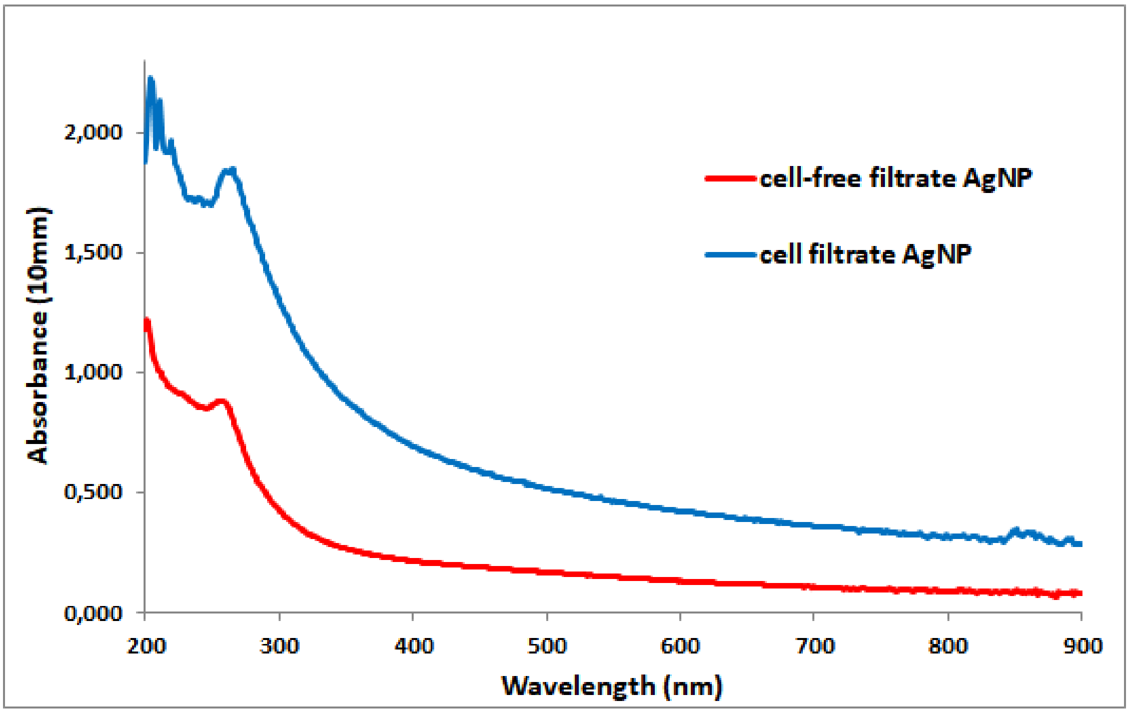

Figure S4. Absorption spectra of silver nanoparticles synthesized using cellular (blue) and cell-free (red) filtrates after six months of storage.
